# Factors associated with the high susceptibility to depression of women during the perimenopause

**DOI:** 10.1002/brb3.2826

**Published:** 2022-12-08

**Authors:** Hongying Han, Xiaowei Xia, Huirong Zheng, Zhiyong Zhong, Chongbang Zhao, Xianglan Wang, Ximei Zhi

**Affiliations:** ^1^ Department of Psychiatry The Third Affiliated Hospital of Sun Yat‐sen University Guangzhou Guangdong Province 510630 P. R. China; ^2^ Guangdong Mental Health Center Guangdong Provincial People's hospital Guangdong Academy of Medical Sciences Affiliated School of Medicine of South China University of Technology Guangzhou Guangdong Province 510080 P. R. China; ^3^ Guangdong Geriatrics Institute Guangdong Provincial People's Hospital Guangdong Academy of Medical Sciences Guangzhou Guangdong Province 510080 P. R. China; ^4^ Department of Psychiatry The Fifth Affiliated Hospital of Sun Yat‐sen University No. 52, East Meihua Road Zhuhai Guangdong Province 519000 P. R. China

**Keywords:** depressive disorders, perimenopause, psychosocial factors, sex hormones, susceptibility

## Abstract

**Objective:**

This study investigated the factors associated with the high susceptibility of perimenopausal women to depression.

**Methods:**

A total of 66 perimenopausal women participated in this study. The Zung self‐rating depression scale (SDS) was used to evaluate the intensity of depressive symptoms. The modified Kupperman index (KI) was used to assess common perimenopausal symptoms. Psychosocial factors were assessed via the Eysenck Personality Questionnaire, attitudes toward menopause checklist, and metacognition questionnaire (MCQ). Levels of serum estradiol, testosterone, and progesterone were determined.

**Results:**

There were statistically significant associations between SDS standard score and the KI scale score (*β* = .361, *p* = .001), years of education (*β* = −.309, *p* = .005), and F3 cognitive self‐consciousness score of MCQ (*β* = −.234, *p* = .032; adjusted *R* ^2^ = .264, *F* = 8.759, *p* < .001).

**Conclusions:**

High susceptibility to depression of perimenopausal women may be related to lower educational level, more severe perimenopausal symptoms, and altered metacognition.

## Graphical abstract

The perimenopause is a window of susceptibility to depression for women. The causes of the high susceptibility of perimenopausal women to depression remain unclear. High susceptibility to depression of perimenopausal women may be related to lower educational level, greater extraversion and unstable personality characteristics, more severe perimenopausal symptoms, and altered metacognition.

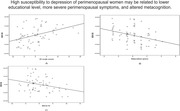

## 1 INTRODUCTION

Women are more susceptible to depressive disorders than men, and the incidence of major depressive disorder in women is twice that in men (Belmaker & Agam, 2008). A wealth of evidence has confirmed perimenopause as a window of vulnerability to depression for women, and the risk of depressive episodes during perimenopause is twice that in premenopausal stages (Freeman, 2010; Parry, 2008). First‐onset or recurrent depressive episodes during perimenopause are referred to as perimenopausal depression (Parry, 2008). The Zung self‐rating depression scale (SDS) is used globally in clinical and research assessments of depressive symptoms and depression intensity (Li et al., 2008). Therefore, we presumed that perimenopausal women with higher SDS scale scores would be more susceptible to perimenopausal depression.

The causes of the high susceptibility of perimenopausal women to depression remain unclear. A multiple factors model has been increasingly and widely accepted, namely, endocrinal changes, perimenopausal symptoms (e.g., hot flushes and night sweating, sleep disturbance, palpitation, vertigo, arthralgia/myalgia, and nervousness), stressful life events, and personal psychosocial features may all be involved (Gibbs et al., 2013). The estrogen withdrawal hypothesis postulates that estrogen insufficiency directly leads to depression (Avis, 2016). Gordon et al. (2015) suggested a novel heuristic model of perimenopausal depression, emphasizing fluctuations in allopregnanolone, and the mediation of γ‐ aminobutyric acid (GABA). However, previous studies of the association between perimenopausal depression and serum sex hormones have shown inconsistent results (Bromberger et al., 2010; Daly et al., 2003; Studd, 2015). The domino hypothesis posits that vasomotor instability results in hot flushes and night sweating; these so‐called vasomotor symptoms (VMS) are features of the perimenopausal stage that later lead to chronic sleep disruption, which in turn lead to irritability and depression (Avis, 2016; de Kruif et al., 2016). However, there exists evidence of no relationship between VMS and depression (Worsley et al., 2014), and the remission of VMS does not consistently relieve depression (Soares et al., 2004). The stress hypothesis supposes that stressful life events play an important role in the occurrence of perimenopausal depression (Schmidt et al., 2004). Further, studies have found that attitudes toward menopause and other health perceptions, and metacognition status, may also be involved in perimenopausal depression (Papageorgiou & Wells, 2003; Yılmaz et al., 2011).

The present study recruited female volunteers from communities via advertisements to avoid systematic bias associated with selecting patients with depressive disorders as subjects. Then, comprehensive data were collected with respect to demographics, depressive symptoms, perimenopausal symptoms, personality characteristics, metacognition, attitudes toward menopause, and serum sex hormones, after which correlation statistics were obtained. Thus, this study provided more evidence regarding the factors associated with the high susceptibility of perimenopausal women to depressive disorders.

## 2 METHODS

### 2.1 Subjects

All subjects were recruited by placing advertisements in the Third Affiliated Hospital of Sun Yat‐sen University and some nearby communities from October 2012 to July 2013. Sixty‐six volunteers participated in this study, all signed informed consent forms (ICF) before the study. The inclusion criteria were as follows: (1) women from 45 to 55 years old, (2) who met the criteria for perimenopause according to the Stages of Reproductive Aging Workshop System (Harlow et al., 2012), (3) with more than 5 years of elementary school education to permit completion of the self‐report scale and ICF, and (4) who were Han Chinese and right‐handed. Exclusion criteria included: (1) A history of medical events that might have significantly affected the study outcome, such as metabolic or endocrine disease; (2) personal or family history of mental disorders; and (3) recent notable negative life events, such as divorce, loss of job, financial issues, and/or death of a family member.

### 2.2 Clinical symptom assessment

The Zung SDS was used to evaluate depressive symptoms and their severity during the previous week. It consists of 20 items rated on a scale from 1 to 4. The raw score consists of the sum of all item scores, and the standard score (SS) is calculated as the raw score × 1.25 (range: 25–100). An SS of 50 has been used as a criterion value for depression in screening work. SS values of <50, 50–59, 60–69, and >70 are typically used to denote depression severity as none, mild, moderate‐to‐severe, and very severe, respectively (Li et al., 2008; Zung et al., 1965).

The modified Kupperman index (KI) was used to evaluate 13 common perimenopausal symptoms. Each symptom was assessed on a four‐point Likert scale (0 = none/no symptom, 3 = severe), and each item was weighted when calculating the total sum score (range: 0–63). Scores 0–6, 7–15, 16–30, and >30 defined the degree of severity as none, mild, moderate, and severe, respectively. These 13 symptoms were classified into 3 subscale factors, namely, urogenital symptoms (including urinary tract infection and sexual complaints), somatic symptoms (including sweating/hot flushes, paresthesia, palpitation, vertigo, headache, formication, and arthralgia/myalgia), and psychological symptoms (including insomnia, fatigue, nervousness, and melancholia) (Kupperman et al., 1953; Tao et al., 2013).

### 2.3 Psychological assessment

The Chinese version of the Eysenck Personality Questionnaire (EPQ) was used to assess the personal characteristics (Gong, 1984) and four dimension scores, namely, extraversion/introversion (E), neuroticism/stability (N), psychoticism/socialization (P), and lying/social desirability (L).

Attitudes toward menopause (ATM) were assessed using the ATM checklist, a self‐rating questionnaire developed by Neugarten et al. (1963) to investigate women's positive and negative ATM. Its reliability and validity have been confirmed in cross‐cultural research (Avis & McKinlay, 1991; Ghaderi et al., 2010; Neugarten et al., 1963). This checklist consists of 34 statement sentences that refer to common beliefs or thoughts about menopause in women, 15 representing positive and 34 negative attitudes. Each item is rated on a 5‐point Likert scale (1 = strongly disagree, 2 = disagree, 3 = do not know, 4 = agree, 5 = strongly agree). The two subscale scores of the positive and negative attitudes were calculated by summing the scores of the responses to each question. Negative items were reverse scored so that higher scores indicated more positive attitudes (Ghaderi et al., 2010; Huffman et al., 2005).

The 30‐item metacognition questionnaire (MCQ‐30) was used to assess 3 domains of positive and negative metacognitive beliefs, metacognitive monitoring, and judgments of cognitive confidence (Wells & Cartwright‐Hatton, 2004). Responses were provided using a four‐point Likert scale (1 = do not agree, 2 = agree slightly, 3 = agree moderately, 4 = agree very much). The scale assesses five factors: cognitive confidence (F1), positive beliefs (F2), cognitive self‐consciousness (F3), uncontrollability and danger (F4), and the need to control thoughts (F5). Each factor score was calculated by summing the item scores of the relevant subscale.

### 2.4 Sex hormone examination

Every subject who met the inclusion criteria had an appointment scheduled specifically according to her menstrual cycle: one of the first 6 days of menstruation for women with identifiable menstrual cycles, and any day at their convenience for women with irregular menstrual cycles (i.e., the interval between menstrual periods of more than 2 months). Subjects’ serum estradiol, testosterone, and progesterone levels were assessed during their appointments. Fasting blood samples were collected from all participants in the early morning. The serum concentrations of these sex hormones were measured using the Siemens ADVIA Centaur XP Immunoassay System (ADVIA Centaur XP, Siemens Healthcare Diagnostics Inc., New York, USA) in the Department of Clinical Laboratories in the Third Affiliated Hospital of Sun Yat‐sen University.

### 2.5 Statistical analysis

All statistics were obtained using the Statistical Package for the Social Sciences (SPSS 26.0; SPSS Inc., Chicago, IL). The simple regression was used to analyze the relationships among the SDS SS and demographic, serum sex hormones, KI scores, EPQ scores, ATM scores, and MCQ scores, respectively. FDR (false discovery rate) approach was used for the multiple testing corrections. Variables with *p* < .2 in the simple regression model were selected as independent variables for the multiple stepwise regression analysis with the dependent variable of SDS SS. The significant level of multiple regression analysis was set as .05.

## 3 RESULTS

### 3.1 SDS assessment

All participants completed the SDS and the mean SS = 44.74, SD = 9.84; range: 26.0–65.0. Regarding depression intensity, 42 (63.64%) persons had no depressive symptoms, 16 (24.24%) reported mild depression, and 8 (12.12%) reported moderate or severe depression.

### 3.2 Simple linear regression analysis with the dependent variable of SDS SS

#### 3.2.1 Demographic data

In total, 66 women participated in this study (mean age = 47.50, SD = 2.01 years, range: 45–52 years). They had an average of 7.78 (SD = 1.83) years of education (range: 5–12). Twenty (30.3%) participants had one child, and 44 (66.7%) had two or more children. Approximately half of the participants had a family income of more than 3000 RMB per month. Sixteen (24.2%) women reported none or mild work pressure, and 5 (7.6%) persons felt severe pressure. Body mass index (BMI) ranged from 19.53 to 32.05 kg/m^2^ with a mean value of 24.24 (SD 2.84) kg/m^2^. According to the results of the simple regressions, variables including education years (*β* = −.320, *p* = .009), BMI (*β* = .253, *p* = .041), and child numbers (*β* = .172, *p* = .168) were selected as an independent variables for the multiple regression. After the FDR correction, SDS SS was negatively related to education years (*q* = .047). See Table 1.

**TABLE 1:** Simple regression models of the self‐rating depression scale (SDS) standardized score (*N* = 66)

	Description	Simple regression model					
		*B*	*β*	Adjusted *R* ^2^	*F*	*p*	*q_FDR_*
Age (years, mean ± SD)	47.50 ± 2.01	−.262	−.056	−.012	.199	.657	.726
Education (years, mean ± SD)	7.78 ± 1.83	−1.726	−.320	.088	7.301	.009	.047
BMI (kg/m^2^, mean ± SD)	24.24 ± 2.84	.876	.253	.049	4.360	.041	.108
Children (number)		3.104	.172	.014	1.944	.168	.321
0	2 (3.0%)						
1	20 (30.3%)						
2 or more	44 (66.7%)						
Monthly incoming (RMB)		1.304	.103	−.005	.691	.409	.573
< 1000	2 (3.0%)						
1000–3000	31 (47.0%)						
3000–5000	23 (34.8%)						
>5000	10 (15.2%)						
Work pressure level		−.461	−.025	−.015	.041	.839	.839
None or mild	16 (24.2%)						
Moderate	45 (68.2%)						
Severe	5 (7.6%)						
Sex hormones (median [min, max])							
Estradiol (pmol/L)	333.92 (43.31, 1507.84)	−.003	−.091	−.007	.523	.468	.578
Testosterone (nmol/L)	0.60 (0.35, 1.82)	−2.904	−.103	−.005	.690	.409	.537
Progesterone (nmol/L)	1.01 (0.48, 46.47)	−.158	−.170	.014	1.911	.172	.301
Modified Kupperman index (mean ± SD)							
Total score	8.91 ± 6.04	.629	.386	.136	11.199	.001	.021
Severity							
No symptom	26 (39.4%)						
Mild	31 (47.0%)						
Moderate‐to‐severe	9 (13.6%)						
Eysenck Personality Questionnaire (mean ± SD)							
E	51.36 ± 9.05	.353	.325	.092	7.564	.008	.056
P	56.14 ± 7.59	−.110	−.085	−.008	.462	.499	.582
N	43.33 ± 9.17	.394	.367	.121	9.942	.002	.021
L	54.09 ± 9.36	−.335	−.319	.088	7.249	.009	.038
Attitudes toward menopause checklist (mean ± SD)							
Positive	48.27 ± 6.93	.173	.119	−.001	.918	.342	.513
Negative	57.35 ± 7.56	−.243	−.181	.018	2.164	.146	.307
Metacognition questionnaire (mean ± SD)							
F1	10.65 ± 3.49	.718	.255	.050	4.433	.039	.137
F2	8.11 ± 3.16	.503	.161	.011	1.708	.196	.317
F3	13.26 ± 4.12	−.608	−.255	.050	4.437	.039	.117
F4	9.61 ± 3.56	.590	.213	.031	3.056	.085	.198
F5	10.98 ± 4.108	−.111	−.046	−.013	.136	.713	.749

Abbreviations: BMI, body mass index; E, extraversion/introversion; F1, cognitive confidence; F2, positive beliefs; F3, cognitive self‐consciousness; F4, uncontrollability and danger; F5, need to control thoughts; FDR, false discovery rate; L, lying/social desirability; N, neuroticism/stability; P, psychoticism/socialization.

#### 3.2.2 Sex hormones

Table 1 shows the serum estradiol, testosterone, and progesterone concentrations. They all were insignificantly correlated with the SDS SS as to the results of the simple regressions, respectively. According to the selective criteria, serum progesterone concentration (*F* = 1.911, *p* = .172) was selected as one of the independent variables for the multiple regression.

#### 3.2.3 KI scores

The positive screening rates (percentages of the item score ≥1) of symptoms from high to low were: 63.6% for Fatigue, 60.6% for arthralgia/myalgia, 57.5% for headache, 54.5% for vertigo, 50.0% for nervousness, 47.0% for sexual complaints, 42.4% for paresthesia, 36.4% for sweating/hot flushes, 28.8% for palpitation, 25.8% for insomnia, 22.7% for formication, 15.2% for melancholia, and 9.1% for urinary tract infection. Symptoms of fatigue, arthralgia/myalgia, headache, vertigo, and nervousness were common, with incidences of more than 50%. Regarding the severity level of KI, 39.4% of participants reported no symptoms, 47.0% reported mild symptoms, and 13.6% reported moderate‐to‐severe symptoms.

As the simple regression results, the total KI score (*β* = .386, *p* = .001, *q_FDR_* = .021) was significantly related to the SDS SS and was selected as an independent variable for the multiple regression. See Table 1.

#### 3.2.4 EPQ, MCQ, and ATM scores

As shown in Table 1, SDS SS was significantly related to scores of E (*β* = .325, *p* = .008), N (*β* = .367, *p* = .002), and L (*β* = −.319, *p* = .009) subscale scores of EPQ; and F1 (*β* = .255, *p* = .039) and MCQ F3 (*β* = −.255, *p* = .039) subscale scores of MCQ. The relationships between SDS SS and ATM negative subscale score (*β* = −.181, *p* = .146), MCQ F2 score (*β* = .161, *p* = .196), and MCQ F4 score (*β* = .213, *p* = .085) were not significant, but these three *p* values were lower than .2. Thus, all these variables were selected as independent variables in the multiple regression analysis. Results of FDR corrections showed that the N score was positively correlated with SDS SS (*q* = .021), and the L score was negatively correlated with SDS SS (*q* = .038).

### 3.3 Multiple linear regression analysis of SDS SS

**FIGURE 1:**
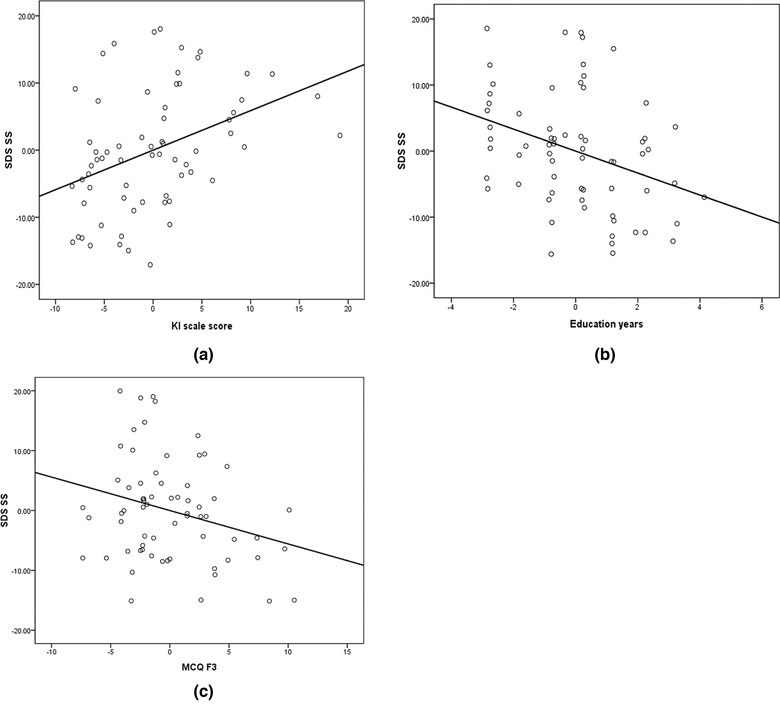
Regression analysis of self‐rating depression scale (SDS) standard score (SS). (a) SDS SS and Kupperman index (KI) scale scores. (b) SDS SS and education years. (c) SDS SS and metacognition questionnaire (MCQ) F3 cognitive self‐consciousness score

A linear multiple stepwise regression analysis was carried out with SDS SS as the dependent variable. The independent variables were years of education; BMI; child numbers; serum progesterone concentration; KI scale scores; negative subscale scores of ATM; E, N, and L subscale scores of EPQ; and F1, F2, F3, and F4 subscale scores of MCQ according to the selective criteria for independent variables. The statistic results showed that SDS SS were significantly related to KI scale scores (*β* = .361, *p* = .001), years of education (*β* = −.309, *p* = .005), and F3 cognitive self‐consciousness scores of the MCQ (*β* = −.234, *p* = .032; overall model, adjusted *R* ^2^ = .264, *F* = 8.759, *p* < .001; see Table 2, Figure 1).

**TABLE 2:** Linear multiple stepwise regression model of self‐rating depression scale (SDS) standard score (SS)

Dependent variable	Independent variables	*B*	*β*	*p*	Adjusted *R* ^2^	*F*	*p*
SDS SS	KI scale score	.589	.361	.001	.264	8.759	<.001
	Education	−1.664	−.309	.005			
	MCQ F3 cognitive self‐consciousness	−.558	−.234	.032			

Abbreviations: KI, Kupperman index; MCQ, metacognition questionnaire.

## 4 DISCUSSION

This study discovered that 12.12% of perimenopausal women had moderate or severe depression, and depression scale scores were related to education level, perimenopausal symptoms, metacognition, and personality characteristics. Depression scores were not significantly related to serum sex hormone levels and ATM.

According to the regression results, lower education level was a risk factor for depression, consistent with previous studies. Lin et al. (2013) investigated 3359 Taiwanese women aged from 40 to 50 years using the Taiwanese Depression Questionnaire and also found a higher percentage of low education level (less than 12 years) in women with depression (88.8%) than in women without depression (82.1%). Bromberger et al. (2010) reported that in their 12‐year community‐based follow‐up cohort study of 3302 women aged from 42 to 52 years, 802 women had high scores (≥16) on the Center for Epidemiologic Studies Depression Scale during the perimenopause, and 70% of them had less than college‐level education, whereas 53% of women without depressive symptoms had education levels lower than college.

The present study revealed significant associations between perimenopausal symptoms and depression. Depressive symptoms were positively related to the urogenital, psychological, and somatic symptoms in perimenopause. Many previous studies have reported relationships between perimenopausal symptoms and depression, but the key symptoms have varied in different studies, for example, hot flushes (Freeman, et al., 2009); hot flushes, sweats, and sleep problems (Burleson et al., 2010); somatic symptoms, including headaches, feeling dizzy or faint, pressure or tightness in the head or body, parts of the body feeling numb or tingly, loss of feeling in hands or feet, breathing difficulty, and muscle and joint pain (Gibbs et al., 2013); and hot flashes or sweats, and dyspareunia or vaginal dryness (Li et al., 2008). In the current study, insomnia, sexual complaints, fatigue, arthralgia/myalgia, and palpitations were more important perimenopausal symptoms with respect to the occurrence of depression than other symptoms. Therefore, the results of this study do not wholly support the domino hypothesis of perimenopausal depression.

It is well known that cognition mediates individual emotional or behavioral responses to stimuli, and depression is relevant to a maladaptive cognitive model, for instance, negative automatic thoughts and dysfunctional schema (Beck, 2002). Wells and Matthews (1996) posited the self‐regulatory executive function (S‐REF) model of emotional disorders, which proposed that patients with depression had a particular cognitive‐attentional “syndrome” consisting of heightened self‐focus, repetitive negative cognitions, maladaptive coping behavior, and threat monitoring, which contribute to emotional disturbance (Papageorgiou & Wells, 2003; Wells & Matthews, 1996). Moreover, on the basis of the theoretical hypothesis, they developed the MCQ instrument for the assessment of metacognition. In the current study, MCQ F3 showed significant associations with SDS SS, and cognitive and anxiety symptoms, which indicates that less monitoring and focusing on cognitive processes were relevant to higher depression susceptibility. The effect of F3 on depression in this study is inconsistent with the S‐REF model. This discrepancy may have occurred due to differences in the study subjects, which in this study were perimenopausal community‐dwelling women but not patients with depression; nor did the current study consider the whole population, including men and all age ranges. Additionally, lack of self‐confidence or self‐efficiency regarding memory (high MCQ F1 score) and excess worry about the uncontrollability of thoughts (high MCQ F4 score) may be related to depressive symptoms. Similar findings were reported by Yılmaz et al. (2011) in voluntary participants and Papageorgiou and Wells (2003) in patients with depression.

Generally, personality characteristics are considered fundamental factors in many mental disorders. The current study found that women with higher EPQ neuroticism (N) and lower lying (L) scores were at a relatively high risk of depression. These findings accord with a study of sex differences in vulnerability to depression that found neuroticism, as evaluated by EPQ, was higher in women than in men and contributed to women's high vulnerability to depression (Carrillo et al., 2004). Similarly, an extensive cross‐sectional survey of 35,832 men (16,104) and women (19,728) aged 20–89 years in Norway found a relationship between depression and both neuroticism and extraversion in the general population (Grav et al., 2012).

The relationship between ATM and depression is inconsistent and controversial (Ayers et al., 2010). The present study did not find significant associations between SDS scores and positive or negative attitudes scores. As the attitudes toward menopause are cultural, it may be helpful for more valuable and exact assessment to develop native questionnaires. At the same time, there were no significant relationships between SDS scores and sex hormones, which may be due to the small sample size or different detection methods from previous studies. Moreover, the results of this study do not support the estrogen withdrawal hypothesis of perimenopausal depression.

The limitations of this study included a relatively small sample size and the intentional exclusion of volunteers with obviously recent stressful events to control for the potential confounds of stress reactions. Thus, future research might target the effects of stressful events on perimenopausal depression.

In summary, the present study's findings provide new evidence for the multiple factors model of the high susceptibility to depression of perimenopausal women. The relevant factors consisted of (1) diathesis factors, including high neuroticism and aberrant metacognitive processes; (2) unfavorable developmental circumstances, namely, limited educational experience in childhood or adolescence; and (3) current harmful somatic complaints associated with severe perimenopausal symptoms. According to the S‐REF model, stressful events in early life foster aberrant metacognition (e.g., negative beliefs or maladaptive cognitive strategies) and dysfunctional schema (Kraft et al., 2017; Wells & Matthews, 1996). The effectiveness of metacognitive therapy (MCT) has been demonstrated in clinical practice (Hagen et al., 2017). Comprehensive interventions may decrease the vulnerability to depressive disorders of women at high risk. Such interventions include health education regarding perimenopause, psychological MCT interventions, cognitive‐behavioral therapy, and other effective psychological therapies; medical treatments for perimenopausal symptoms may also be helpful.

## 5 CONCLUSION

The high susceptibility to the depression of perimenopausal women was associated with aberrant metacognitive features, lower educational levels, and more severe perimenopausal symptoms.

## AUTHOR CONTRIBUTIONS

Hongying Han, Xiaowei Xia, Xianglan Wang, and Ximei Zhi; designed the study and wrote the protocol. Hongying Han and Xiaowei Xia; managed the literature searches and analyses. Huirong Zheng, Zhiyong Zhong, and Chongbang Zhao; helped to gather research data, and Hongying Han wrote the first draft of the manuscript. All authors contributed to and approved the final manuscript.

## CONFLICT OF INTEREST

The authors declare that the research was conducted in the absence of any commercial or financial relationships that could be construed as a potential conflict of interest.

### PEER REVIEW

The peer review history for this article is available at https://publons.com/publon/10.1002/brb3.2826.

## Data Availability

The raw data supporting the conclusions of this manuscript will be made available by the authors, without undue reservation, to any qualified researcher.
